# E-cadherin as a prognostic indicator in primary breast cancer

**DOI:** 10.1054/bjoc.2001.2178

**Published:** 2001-12

**Authors:** C Parker, R S Rampaul, S E Pinder, J A Bell, P M Wencyk, R W Blamey, R I Nicholson, J F R Robertson

**Affiliations:** Departments of Histopathology, Surgery, University Of Nottingham, City Hospital, Hucknall Road, Nottingham NHS Trust; Tenovus Institute, Cardiff

**Keywords:** Breast cancer, immunohistochemistry, cadherin, prognosis

## Abstract

Epithelial cadherin (E-CD) is a member of the cadherin family of cell adhesion molecules and has been implicated as an invasion suppressor molecule in vitro and in vivo. We analysed 174 breast tumours from the Nottingham/Tenovus Breast Cancer Series immunohistochemically for E-CD expression using the mouse monoclonal antibody HECD-1 (Zymed Laboratories Inc.). In normal epithelial cells E-CD was strongly expressed at cell–cell boundaries. 66% of the breast cancers examined had reduced intensity of E-CD expression with 74% having significant reductions in the proportion of E-CD-positive tumour cells. Using a combined intensity/proportion score, significant associations were found between E-CD expression and tumour type (*P* ≤ 0.001). ER status (*P* = 0.026) and histological grade (*P* = 0.031). Expression of E-CD was not found to be related to recurrence, distant metastases, lymph node stage, vascular invasion, primary tumour size, prognostic group or survival. Thus E-CD expression in human breast cancer appears to have minimal prognostic value, but may have a role as a phenotypic marker. © 2001 Cancer Research Campaign http://www.bjcancer.com


*British Journal of Cancer* (2001) **85**(12), 1958–1963
© 2001 Cancer Research Campaign
doi: 10.1054/ bjoc.2001.2178, available online at http://www.idealibrary.com on IDEAL
http://www.bjcancer.com

### E-cadherin as a prognostic indicator in primary breast cancer

**C Parker[1], RS Rampaul[2], SE Pinder[1], JA Bell[1], PM Wencyk[1], RW Blamey[2], RI Nicholson[3], JFR Robertson[2] and IO Ellis[1]**

[1]Departments of Histopathology and [2]Surgery, University Of Nottingham, City Hospital, Hucknall Road, Nottingham NHS Trust; and [3] Tenovus Institute, Cardiff

**Summary** Epithelial cadherin (E-CD) is a member of the cadherin family of cell adhesion molecules and has been implicated as an invasion suppressor molecule in vitro and in vivo. We analysed 174 breast tumours from the Nottingham/Tenovus Breast Cancer Series immunohistochemically for E-CD expression using the mouse monoclonal antibody HECD-1 (Zymed Laboratories Inc.). In normal epithelial cells E-CD was strongly expressed at cell–cell boundaries. 66% of the breast cancers examined had reduced intensity of E-CD expression with 74% having significant reductions in the proportion of E-CD-positive tumour cells. Using a combined intensity/proportion score, significant associations were found between E-CD expression and tumour type ($P \leq 0.001$). ER status ($P = 0.026$) and histological grade ($P = 0.031$). Expression of E-CD was not found to be related to recurrence, distant metastases, lymph node stage, vascular invasion, primary tumour size, prognostic group or survival. Thus E-CD expression in human breast cancer appears to have minimal prognostic value, but may have a role as a phenotypic marker. © 2001 Cancer Research Campaign http://www.bjcancer.com

**Keywords**: Breast cancer; immunohistochemistry; cadherin; prognosis

Predicting disease outcome is becoming increasingly important in understanding the natural history of breast cancer, planning treatment strategies and counselling the patient. Assessment of certain prognostic 'markers' or 'factors' is now routine in the pathological examination of breast tumours, in order to give an indication of suitability of certain forms of treatment, the risk of recurrence and expected length of survival (Royal College of Pathologist Working Group, 1991). Some of the more traditional markers include histological typing and grading, primary tumour size, lymph node stage, vascular invasion and excision margin status. Numerous putative novel prognostic markers have been identified in recent years through increasing understanding of the biology of breast cancer. One group of these possible factors is the Cadherin family.

Epithelial cadherin (E-cadherin) is a member of the cadherin family of cell adhesion molecules, joining adjacent epithelial cells together via a $Ca^{2+}$-dependent homophilic binding mechanism (Rasbridge et al, 1993). The cytoplasmic domain of E-cadherin (E-CD) complexes with cytoplasmic proteins known as catenins, which links the cadherin with the cytoskeleton and other transmembrane proteins (Kemler, 1993). E-CD mutants with deletion of the catenin-binding domain cannot bind cells together, so catenins are crucial to cadherin function (Kemler, 1993; Hashizume et al, 1996). Cadherins are also considered to be of importance in embryonic development and maintenance of adult tissue architecture, as inactivation of other adhesion systems has little impact if cadherins are fully functional (Van der Wurff et al, 1992; Siitonen et al, 1996).

Several lines of evidence suggest that loss of cell–cell adhesion contributes to the detachment of tumour cells and allows them to transgress normal barriers and migrate to distant sites. To that end collagen invasion assays have shown that cells expressing E-CD were non-invasive, whereas E-CD-negative cells were invasive. Down-regulation of E-CD protein by antisense RNA induced invasive behaviour in previously non-invasive cells (Behrens, 1993). In addition, a transgenic mouse model of pancreatic β cell carcinoma revealed that loss of E-CD expression coincided with transition from well differentiated adenoma to invasive carcinoma (Perl et al, 1998). However, some studies have indicated that the loss of E-CD is reversible, for example in *ras*-transformed Madin Darby Canine kidney cells in nude mice, possibly due to interaction with the host environment (Mareel et al, 1991). Dysfunction of α-catenin has also been implicated. Shimoyama et al (1992) found that a human lung cancer cell line strongly expressed E-CD, but showed reduced E-CD adhesive function. This cell line did not express α-catenin. Some experiments have also indicated that the function of E-CD can be regulated by external factors. For example, tamoxifen restored E-CD function in the MCF-7 breast cancer cell line and inhibited invasion (Bracke et al, 1994).

In the breast, several studies have reported a difference in E-CD expression in carcinoma compared to normal tissue. E-CD expression in ductal carcinoma in situ (DCIS) is similar to that of normal tissue, while invasive ductal carcinomas may have reduced or heterogeneous expression. In contrast, infiltrating lobular carcinoma (ILC) has significantly reduced or absent expression (Gamallo et al, 1993; Oka et al, 1993; Rasbridge et al, 1993; Birchmeier and Behrens, 1994; Lipponen et al, 1994; Siitonen et al, 1996; Charpin et al, 1997; Gonzalez et al, 1999). The reduced expression seen in ILC is assumed to be reflected in the different pattern of invasion compared to ductal and special types of breast cancer.

A number of studies have found correlations between reduced E-CD levels and certain features, such as high grade, loss of oestrogen receptor (ER) and progesterone receptor (PR), axillary

*Received 20 September 2001*
*Accepted 20 September 2001*

*Correspondence to:* IO Ellis node metastases, recurrence, mutated p53 and growth fraction (Siitonen et al, 1996; Hashizume et al, 1996; Charpin et al, 1997; Gupta et al, 1997). Some (Gamallo et al, 1993; Lipponen et al, 1994; Gonzalez et al, 1999) have observed a relationship with histological grade and tumour type, but no other features. Maguire et al (1997), detecting E-CD by ELISA found no relationship with tumour size, node status or ER, but did associate early recurrence with lower E-CD levels. Siitonen et al (1996) proposed that E-CD expression was directly related to progression. Many studies have concluded that E-CD alteration appears to be involved more in invasion and less with metastatic potential than previously suggested and that perhaps other factors, in addition to loss of cell adhesion, are involved in the complex process of metastasis.

In this study we have examined the expression of E-CD in a consecutive series of patients with well characterised primary breast cancer and long-term clinical follow up to provide data on relationships between E-CD expression, primary tumour characteristics and behaviour.

### METHODS

### Patients

A cohort of patients presenting consecutively with primary operable breast cancer was selected randomly from archives of the Nottingham/Tenovus Primary Breast Cancer Series. All patients were treated by a single surgeon (RW Blamey, Nottingham City Hospital), by mastectomy or wide local excision but in this historical series received no adjuvant therapy. Loco-regional lymph nodes were sampled. Patients were reviewed at 3 monthly intervals for 18 months after surgery, then 6 monthly for the next 5 years and yearly thereafter. Data on recurrence, distant metastases and survival, menopausal status, histological grade (Elston and Ellis, 1991), tumour type (Ellis et al, 1992), vascular invasion (Pinder et al, 1994), age of patient at diagnosis, primary tumour size, prognostic group (Pereira et al, 1995) and ER status was recorded and stored on a computer database.

This study included 174 primary operable breast cancer cases, with age of patient ranging from 28–70 years (mean 55 years). Follow up ranged from 7 to 205 months (median 102 months).

### Materials

Immunohistochemistry was performed on paraffin-embedded tissue sections, using the standard streptavidin–biotin–peroxidase complex method. The monoclonal mouse anti-E-cadherin antibody (HECD-1) was supplied by Zymed Laboratories Inc (San Francisco, California, USA). The StreptABC kit used to detect the primary antibody was obtained from Dako Ltd (High Wycombe, Buckinghamshire, UK). The DAB developing solution and the phosphate-citrate buffer with sodium perborate used to dilute the DAB were purchased from Sigma-Aldrich Co Ltd (Poole, Dorset, UK). The 3-aminopropyl-triethoxysilane (TESPA) coating on the microscope slides was also supplied by Sigma-Aldrich Co Ltd.

The primary antibody was diluted to 1 in 400 by a 1/5 solution of NSS (normal swine serum in tris-buffered saline). Tissue sections were treated by microwave retrieval prior to staining. Known positive controls (composite breast tissue) were included with each batch to ensure interbatch reproducibility. Portions of normal tissue within the tumour sections provided internal controls. Negative controls consisted of sections not incubated with primary antibody.

### Assessment of staining

The approximate intensity of E-CD membrane staining of tumour cells was classified as absent (0), weak, but focal (1), weak (2) or strong (3). The weak, but focal, classification was added following pilot studies showing a small number of sections had small weak areas of staining in an otherwise negative tumour. The approximate fraction of tumour cells with membrane staining was also evaluated and divided into 4 categories 0%, less than 25%, 25–75% and greater than 75%.

The 174 tumours were analysed at least twice, until intraobserver agreement was reached and agreement achieved in difficult cases by consensus following assessment by a second observer. All the data obtained (E-CD assessment and patient information) was analysed on an Apple Macintosh computer, using the Statview 4.1 programme. Statistical significance was calculated using the $\chi^2$ tests and Fisher's exact test where appropriate. Survival analysis was performed by applying the log-rank test of significance to Kaplan–Meier cumulative survival curves.

### RESULTS

### Assessment of staining

In both positive controls and internal areas of normal breast tissue the immunostaining was clear around the membrane with minimal background reactivity (Figure 1). The membranes of epithelial cells lining the ducts and lobules of normal breast tissue were strongly and uniformly stained. Myoepithelial cells exhibited weaker staining. E-CD expression could be extremely variable within the same tumours, with differing levels of intensity throughout some tumours. In such cases, the predominant pattern was recorded. Variation in the proportion of cells stained was also occasionally seen, with negative areas interspersed in otherwise positive tumours. Small islands of weakly stained cells were also observed in otherwise negative tumours. Only membrane staining was evaluated, but it was noticed that a significant number of tumours (especially those with weaker staining) had diffuse staining within the cytoplasm. In a very small proportion of cases, staining was prominent around the nucleus of the tumour cell.

### Distribution of E-cadherin expression and analysis of staining

Proportion and intensity were scored separately. In order to further examine E-CD expression and to determine whether there was an

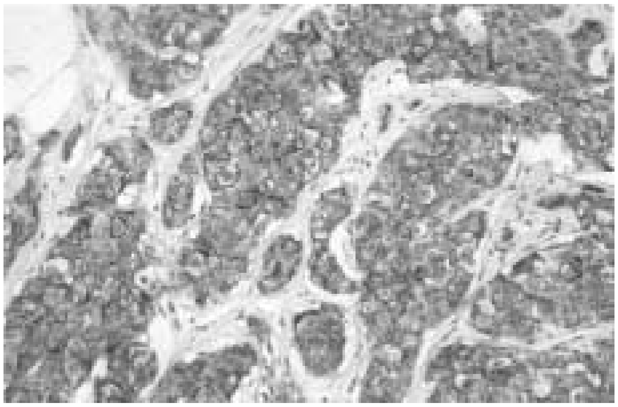

**Figure 1** Photomicrograph of a E-CD positive primary breast cancer

© 2001 Cancer Research Campaign

**Table 1** Patterns of expression of E-cadherin intensity and proportion

| Proportion | 0% | < 25% | 25–50% | > 75% | Total |
|---|---|---|---|---|---|
| Intensity | | | | | |
| 0 | **18** | 0 | 0 | 0 | 18 |
| 1 | 0 | **13** | 0 | 0 | 13 |
| 2 | 0 | **12** | **52** | **20** | 84 |
| 3 | 0 | 5 | **28** | **26** | 59 |
| Total | 18 | 30 | 80 | 46 | 174 |

$\chi^2 = 248.1$, $P < 0.001$.

**Table 2** Method of assignment of overall score for E-cadherin positivity

| Proportion<br>Intensity | | 0<br>(0%) | 1<br>(< 25%) | 2<br>(25–50%) | 3<br>(> 75%) |
|---|---|---|---|---|---|
| Combined | 0 | 0,0 | | | |
| Proportion | 1 | | 1,1 | | |
| Intensity | 2 | | 2,1 | 2,2 | 2,3 |
| Score | 3 | | | 3,2 | 3,3 |

Key to intensity/proportion groups when grouped together (combinations in brackets are derived from the numbers in bold in Table 1):
(0,0) = 0 (negative)
(1,1/2,1/2,2) = 1 (weak and heterogenous)
(2,3) = 2 (weak and homogenous)
(3,2) = 3 (strong and heterogenous)
(3,3) = 4 (strong and homogenous).

**Table 3** Results of univariate analysis. E-cadherin vs other tumour and patient variables

| | $\chi^2$ | *p* |
|---|---|---|
| Variable (#cases) | | |
| Alive (93) vs Dead (73) | 5.14 | 0.273 |
| Local recurrence | | |
| Absent (97) vs Present (46) | 8.43 | 0.077 |
| Regional recurrence | | |
| Absent (93) vs Present (58) | 1.35 | 0.853 |
| Distant metastasis | | |
| Absent (77) vs Present (90) | 2.50 | 0.645 |
| Menopausal status | | |
| Pre- (53) vs Post (120) | 5.42 | 0.247 |
| Age | | |
| < 40 (13) vs 40–49(41) vs 50 or more (119) | 6.39 | 0.604 |
| Size | | |
| 10 mm or less (14) vs 11–20 (85) vs 21–30 (54) vs > 30 (14) | 9.79 | 0.634 |
| Grade | | |
| 1 (31) vs 2 (69) vs 3(66) | 16.92 | 0.031 |
| Lymph node status | | |
| Negative (98) vs Positive (69) | 2.14 | 0.711 |
| Vascular invasion | | |
| Absent (109) vs Present (30) | 3.37 | 0.499 |
| Type | | |
| Lobular (38) vs other (129) | 30.60 | < 0.001 |
| ER | | |
| ER pos (< 10 fmol $mg^{-1}$ prot) (70) vs ER neg (10 fmol $mg^{-1}$ prot) (59) | 11.04 | 0.026 |

association with biological and clinical variables, a combined score of intensity and proportions was derived (range 0–4) (Tables 1 and 2). Using this approach, cases were placed into 4 distinct groups showing no reactivity (0), weak and heterogeneous (1), weak and homogeneous (2), strong and heterogeneous (3) and strong and homogeneous (4) expression. The majority of (66%) tumours had some reduction in expression, but few were completely absent. 74% showed reduced expression when compared to the strong and uniform levels of expression seen in normal epithelium.

**Table 4** Correlation histological grade

| Intensity/<br>Proportion | | 0<br>No. (%) | 1<br>No. (%) | 2<br>No. (%) | 3<br>No. (%) | 4<br>No. (%) | Total<br>No. |
|---|---|---|---|---|---|---|---|
| Grade | 1 | 6 (19) | 16 (52) | 0 (0) | 4 (13) | 5 (16) | 31 |
| | 2 | 8 (12) | 30 (43) | 7 (10) | 10 (14) | 14 (20) | 69 |
| | 3 | 4 (6) | 26 (39) | 11 (17) | 19 (29) | 6 (9) | 66 |
| Total | | 18 | 72 | 18 | 33 | 25 | 166 |

$\chi^2 = 16.92$, $P = 0.031$.
Intensity/Proportion: 0 – negative,
1 – weak and heterogeneous,
2 – weak and homogeneous,
3 – strong and heterogeneous,
4 – strong and homogeneous.

**Table 5** Correlation with ER status

| Intensity/<br>Proportion | 0<br>No. (%) | 1<br>No. (%) | 2<br>No. (%) | 3<br>No. (%) | 4<br>No. (%) | Total |
|---|---|---|---|---|---|---|
| ER negative | 4 (6) | 36 (51) | 7 (10) | 18 (26) | 5 (7) | 70 |
| ER positive | 8 (14) | 21 (36) | 8 (14) | 9 (15) | 13 (22) | 59 |
| Total | 12 | 57 | 15 | 27 | 18 | 129 |

$\chi^2 = 11.04$, $P = 0.026$.
Intensity/Proportion: 0 – negative,
1 – weak and heterogeneous,
2 – weak and homogeneous,
3 – strong and heterogeneous,
4 – strong and homogeneous.

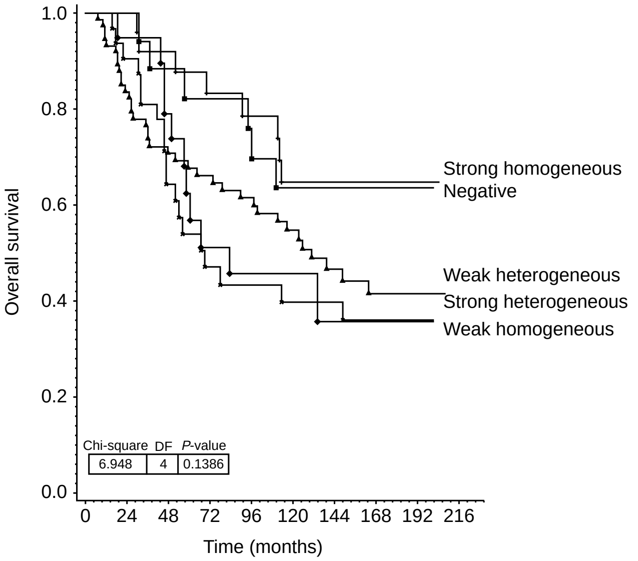

**Figure 2** Graph showing overall survival by E-CD intensity/proportion

 *© 2001 Cancer Research Campaign*

Analysis of intensity/proportion scores with other variables (Table 3) showed only grade ($P$ = 0.031), tumour type ($P$ < 0.001) (Table 4) and ER ($P$ = 0.026) (Table 5) were associated with E-CD immunoreactivity. There was no statistical difference between overall survival of E-CD-positive and -negative patients (Figure 2).

### DISCUSSION

Previous studies of E-cadherin expression in breast and other cancers have varied widely in patient samples and scoring methods, which make comparisons difficult. Most series (whether using frozen or fixed tissue), including the present one, have shown that the epithelial cells of normal ducts stain strongly and clearly around the membrane (Gamallo et al, 1993; Moll et al, 1993; Rasbridge et al, 1993; Birchmeier and Behrens 1994; Hashizume et al, 1996; Gupta et al, 1997; Hunt et al, 1997). Oku et al (1993) noted that 80% of frozen tumour sections with reduced E-CD expression were heterogeneous (Oka et al, 1993). Charpin's study (Charpin et al, 1997), also on frozen sections, noted that tumours with weaker E-CD expression were heterogeneous. Studies on paraffin-embedded tumours, as in this study, have also observed distinct intratumoural variation in staining intensity (Lipponen et al, 1994).

The diffuse cytoplasmic staining seen in this study has been mentioned in other studies. Charpin et al (1997) and Siitonen et al (1996) using frozen section material found that cytoplasmic staining was present in some lobular-type tumours. We did not evaluate cytoplasmic staining in addition to membrane staining, as it was assumed that any E-CD protein present in the cytoplasm was non-functional. However, other studies have described E-CD cytoplasmic staining based on the hypothesis that it may be new protein stored in the Golgi apparatus or internalised for some reason (Rasbridge et al, 1993). Alternatively, as cytoplasmic staining tends to be seen in tumours with some loss of E-CD expression, there may be a defect in transport mechanisms moving the protein to the membrane. A further possibility involves the abnormal localisation or disruption of α-catenin, which may fail to anchor the protein in the membrane.

Direct comparisons of frequency distributions are not possible due to differences in evaluating E-CD expression in different series. Most studies report reductions in E-CD expression in a significant number of breast carcinomas, ranging from 45% to 63% of cases (Oka et al, 1993; Rimm et al, 1995; Siitonen et al, 1996). However, the majority of the above studies were performed on frozen tissue sections where staining is weaker than in paraffin-embedded tissues (Lipponen et al, 1994). It appears that complete loss of E-CD is uncommon, but the majority of tumours experience some reduction in E-CD expression.

No correlations between E-CD expression and the following features were found in this series: local recurrence, regional recurrence, distant metastases, lymph node status, vascular invasion, tumour size, prognostic group or overall survival. There are conflicting reports in the literature regarding the relationship of E-CD expression with other prognostic factors and pathological features, but local recurrence, vascular invasion and prognostic group have not been reported as having any association with E-CD. Siitonen et al (1996) and Oka et al (1993) found a correlation between loss of E-CD and the presence of nodal metastases, but this has not been widely reported (Gamallo et al, 1993; Lipponen et al, 1994; Charpin et al, 1997; Maguire et al, 1997). Oka et al (1993) found an association between E-CD expression and metastatic disease on frozen sections. Many studies do not have data available on the development of distant metastases in patients with primary breast carcinomas and further investigation is required. One study (Oka et al, 1993) observed a relationship between E-CD and tumour size. Our study, and most others have not found any correlation (Gamallo et al, 1993; Lipponen et al, 1994; Siitonen et al, 1996; Charpin et al, 1997; Maguire et al, 1997; Gonzalez et al, 1999).

As in this series of paraffin-embedded material, several studies on frozen section material have reported a correlation between reduced E-CD expression and high histological grade (Gamallo et al, 1993; Moll et al, 1993; Oka et al, 1993; Siitonen et al, 1996; Charpin et al, 1997; Gonzalez et al, 1999). Others, also using paraffin-embedded tissue, have however failed to find such a correlation (Lipponen et al, 1994). Whilst the difference in methodology may account for the discordance, more studies using paraffin-embedded sections are clearly required. It is interesting however, that when the data was analysed as a combined intensity/proportion score, histological grade was statistically significantly ($P$ = 0.031) associated with E-CD immunohistochemical expression. If, however, analysed independently as either intensity or proportion alone there was no association found (data not shown). Thus it is possible that this combined approach may provide a more meaningful analysis of E-CD expression.

Oestrogen is thought to regulate the expression of E-CD (Oka et al, 1993). Indeed E-CD is not expressed in vitro in hormone independent systems (Siitonen et al, 1996). If oestrogen enhances expression of E-CD, loss of circulating oestrogen after the menopause may result in reduction of E-CD to a certain extent. However, in this study, menopausal status was not significantly related to E-CD expression ($P$ = 0.247), possibly as there was a greater proportion of invasive lobular tumours (which are likely to be E-CD negative) in postmenopausal women.

A particularly interesting finding in this series was the relationship between the proportion of E-CD-positive cells and the ER status of the tumour (Table 5). ER-positive tumours tended to have 'all or nothing' E-CD expression and were predominantly in the groups showing homogeneous staining either completely negative, weak homogeneous or, more commonly, strong homogeneous reactivity (scores 0, 1 or 4). ER-negative tumours had predominately heterogeneous expression, mostly a weak heterogeneous (score 1) or strong heterogeneous (score 3) pattern of immunopositivity being seen. Literature concerning the relationship between E-CD expression and ER status is contradictory, with several studies finding a correlation and yet others no association; our data indicate that this may be related to the methodology of assessment of positivity. Some studies have found that loss of E-CD is associated with loss of ER (Moll et al, 1993; Siitonen et al, 1996; Charpin et al, 1997). These studies were all performed on frozen tissue, while Lipponen's work (Lipponen et al, 1994) using paraffin-embedded tissue noted a correlation in the opposite orientation (i.e. reduced E-CD was found in ER-positive tumours). In the present study ER-positive tumours tended to be strongly homogeneously positive compared to ER-negative tumours (22% vs 7%). However ER-positive tumours were also more commonly completely negative than those which were ER-negative (14% vs 6%). The relationship between E-CD and ER status may be influenced and indeed confounded by tumour type; ER tends to be retained in lobular cancers and as many of the E-CD-negative tumours are of lobular morphology, many will contain ER. The correlation between ER and E-CD warrants further evaluation.

The loss of E-CD expression in lobular cancers is well documented in the literature and the present study also found that lobular cancers were more likely to be E-CD negative. Classical

*© 2001 Cancer Research Campaign* 

lobular cancers tended to be almost always negative while lobular-mixed types had staining in the mixed areas rather than the foci of classical lobular architecture and morphology. This is consistent with results obtained by Moll et al (1993). Cases of medullary and mucinous carcinoma and DCIS were all found to be E-CD positive, which is supported by Lipponen et al (1994). Most studies, including the present, have reported that invasive ductal carcinomas were mainly E-CD positive, but expression was on occasions reduced. The loss of expression in lobular carcinomas is likely to be reflected in its distinct growth and invasion pattern. Lobular cancers tend to grow infiltratively, as opposed to the expansive pattern seen in many other cancers. Loss of E-CD could lead to 'looser' intercellular connections, so cells can dissociate from each other and invade in strands or clumps, resulting in the characteristic 'Indian file' morphology seen histologically.

E-cadherin expression is regulated through many mechanisms such as gene mutation (Berx et al, 1996), methylation of its promoter sequence (Hiraguri et al, 1998) as well as small G proteins, DNA polymorphism and proteolysis. Recently 11 novel tumour-associated E-CD mutations have been identified (Becker et al, 1999). This work has confirmed that E-CD mutations are associated with morphological subtypes of gastric and breast cancers; specifically diffuse-type gastric cancers and invasive lobular of breast. Tumour types differ in their prognosis, with classical lobular being slightly more favourable than mixed lobular types (Pereira et al, 1995), but the prognosis of tumour type does not appear to be related to E-CD. Tubular and mucinous types are E-CD positive and have excellent prognoses, but medullary tumours also tend to be positive, yet have an average prognosis (Ellis et al, 1992). Ductal carcinomas have poor prognosis, but are mainly E-CD positive. In this context, evaluating E-CD expression may be of some value in distinguishing between pure lobular and mixed variants, as they are sometimes difficult to identify; mixed portions will stain for E-CD.

The use of immunohistochemistry does present some drawbacks in that staining interpretation may be somewhat subjective. It is also difficult to compare different series with different cut-offs for positivity and negativity. Immunohistochemistry in general does not determine if the protein being detected is functional or mutated. E-CD is only one part of a complex system of cell adhesion and genetic alterations in any component of this cadherin–catenin complex can induce loss of adhesion function. However, assessment of immunohistochemistry is the most readily available technique for assessment of historical, archival paraffin-embedded material in any worthwhile numbers. Thus it is only with evaluation using this method that tumours from patients with long-term follow up can be examined at present. Nevertheless, it appears unlikely from these results that loss of E-CD is a key event in the initiation of invasion (Moll et al, 1993; Lipponen et al, 1994). Additional factors, for example scatter factor, may act in combination to damage cell adhesion and aid tumour cell detachment and invasion (Meiners et al, 1998). In conclusion E-CD appears to be of no significant prognostic value in predicting long-term outcome of women with breast cancer in this series.

### REFERENCES

Becker KF, Reich U, Schott C, Becker I, Berx G, van Roy F and Hofler H (1999) Identification of eleven novel tumour-associated E-cadherin mutations. Mutations in brief no. 215. Online. *Human Mutations* **13**: 171

Behrens J (1993) The role of cell adhesion molecules in cancer invasion and metastasis. *Breast Cancer Res Treat* **24**: 175–184

Berx G, Cleton-Jansen AM and Strumane K (1996) E-cadherin is inactivated in a majority of invasive human lobular breast cancers by truncation mutations throughout its extracellular domain. *Oncogene* **13**: 1919–1925

Birchmeier W and Beherns J (1994) Cadherin expression in carcinomas: Role in the formation of cell junctions and the prevention of invasiveness. *Biochim Biophys Acta* **1198**: 11–26

Bracke ME, Charlier C, Bruyneel EA, Labit C, Mareel MM and Castronovo V (1994) Tamoxifen restores the E-Cadherin function in Human Breast Cancer MCF-7/6 Cells and Suppresses Their Invasive Phenotype. *Cancer Research* **54**: 4607–4609

Bussemakers MJG, Giroldi LA, Van Bokhoven A and Schalken JA (1994) Transcriptional regulation of the human E-cadherin gene in human prostate cancer cell lines: Characterisation of the human E-Cadherin gene promoter. *Biochem Biophy Res Comm* **203**(**2**): 1284–1291

Charpin C, Garcia S, Bouvier C, Devictor B, Andrac L, Choux R and Lavaut M (1997) E-Cadherin quantitative immunocytochemical assays in breast carcinomas. *J of Path* **181**: 294–300

Doroudi S, Sheffield JP, Poulsom R, Northover JMA and Hart IR (1993) E-Cadherin expression in colorectal cancer: An immunocytochemical and *In Situ* hybridisation study. *Am J of Pathol* **142**: 981–986

Ellis IO, Galea M, Broughton N, Locker A, Blamey RW and Elston CW (1992) Pathological prognostic factors in breast cancer. II. Histological types. Relationship with survival in a large study with long term follow up. *Histopathology* **20**: 479–489

Elston CW and Ellis IO (1991) Pathological prognostic factors in breast cancer. I. The value of histological grade in breast cancer: experience from a large study with long-term follow-up. *Histopathology* **19**: 403–410

Gamallo C, Palacios J, Suarez A, Pizarro A, Navarro P, Quintanilla M and Cano A (1993) Correlation of E-cadherin expression with differentiation grade and histological type in breast cancer. *Am J of Pathol* **142**: 987–993

Gonzalez MA, Pinder SE, Wencyk P, Bell JA, Elston CW, Nicholson RI, Robertson JFR, Blamey RW and Ellis IO (1999) An immunohistochemical examination of the expression of E-Cadherin, a-and b/y -Catenins, and a2 and b1-Integrins in invasive breast cancer. *J of Pathol* **187**: 523–529

Gupta SK, Douglas-Jones AG, Jasani B, Moragn JM, Pignatelli M and Mansel RE (1997) E-Cadherin expression in duct carcinoma *In Situ* (DCIS) of the breast. *Virchows Archives* **430**: 23–28

Hashizume R, Koizumi H, Ihara A, Ohta T and Uchikoshi T (1996) Expression of β-catenin in normal breast tissue and breast carcinoma: A comparative study with epithelial cadherin and α-catenin. *Histopathology* **29**: 139–146

Hiraguri S, Godfrey T, Nakamura H et al (1998) Mechanisms of inactivation of E-Cadherin in breast cancer cell lines. *Cancer Res* **58**: 1972–1977

Hunt NCA, Douglas-Jones AG, Jasani B, Morgan JM and Pignatelli M (1997) Loss of E-Cadherin expression associated with lymph node metastases in small breast carcinomas. *Virchows Archives* **430**: 285–289

Kemler R (1993) From cadherins to catenins: cytoplasmic protein interactions and regulation of cell adhesion. *Trends in Genetics* **9**(**9**): 317–321

Lee SM (1996) H-Cadherin, a novel cadherin with growth inhibitory functions and diminished expression in human breast cancer. *Nature Medicine* **2**(**7**): 776–782

Lipponen P, Saarelainen E, Ji H, Aaltomaa S and Syrjanen K (1994) Expression of E-Cadherin as related to other prognostic factors and survival in breast cancer. *J of Pathol* **174**: 101–109

Maguire TM, Shering SG, McDermott EW, O'Higgins N, Fenelly JJ, Crown J and Duffy MJ (1997) Assay of E-Cadherin by ELISA in Human Breast Cancers. *European J of Cancer* **33**(**3**): 404–408

Mareel MM, Behrens J, Birchmeier W, De Bruyne G, Vleminikx K, Hoogewus A, Fiers W and Van Roy FM (1991) Down-regulation of E-Cadherin expression in madin darby canine kidney cells inside tumours of nude mice. *Intern J of Cancer* **47**: 922–928

Mayer B, Johnson JP, Leitl F, Jauch KW, Heiss MM, Schildberg FW, Birchmeier W and Funke I (1993) E-Cadherin expression in primary and metastatic gastric cancer: down-regulation correlates with cellular dedifferentiation and glandular disintegration. *Cancer Research* **53**: 1690–1695

Meiners S, Brinkmann V, Naundorf H and Birchmeier W (1998) Role of morphogenetic factors in metastasis of mammary carcinoma cells. *Oncogene* **16**: 9–20

Moll R, Mitze M, Frixen UH and Birchmeier W (1993) Differential loss of E-Cadherin expression in infiltrating ductal and lobular breast carcinomas. *Am J of Pathol* **143**: 1731–1742

Oka H, Shiozaki H, Kobayashi K, Inoue M, Tahara H, Kobayashi T, Takatsuka Y, Matsuyoshi N, Hirano S, Takeichi M and Mori T (1993) Expression of E-Cadherin cell adhesion molecules in human breast cancer tissues and its relationship to metastasis. *Cancer Research* **53**: 1696–1701

Pererria H, Pinder SE, Sibbering DM et al (1995) Pathological prognostic factors in breast cancer IV. Should you be a typer or grader? A comparative study of

 *© 2001 Cancer Research Campaign*

two prognostic variables in operable breast cancer. *Histopathology* **27**: 219–226

Perl A-K, Wilgenbus P, Dahl U, Semb H and Christofori G (1998) A causal role for E-Cadherin in the transition from adenoma to carcinoma. *Nature* **392**: 190–193

Pinder SE, Ellis IO, Galea M, O'Rouke S, Blamey RW and Elston CW (1994) Pathological prognostic factors in breast cancer. III. Vascular invasion: relationship with recurrence and survival in a large study with long-term follow-up. *Histopathology* **24**: 41–47

Pizarro A, Benito N, Navarro P, Palacios J, Cano A, Quintanilla M, Contreras F and Gamallo C (1994) E-Cadherin expression in basal cell carcinoma. *Brit J of Cancer* **69**: 157–162

Rasbridge SA, Gillet CE, Sampson SA, Walsh FS and Millis RR (1993) Epithelial (E-) and placental (P-) cadherin cell adhesion molecule expression in breast carcinoma. *J of Pathol* **169**: 245–250

Rimm DL, Sinard JH and Morrow JS (1995) Reduced α-catenin and E-cadherin expression in breast cancer. *Laboratory Investigation* **72(5)**: 506–512

Royal College of Pathologists' Working Group (1991) Pathology reporting in breast cancer screening. *J Clin Path* **44**: 710–725

Schipper JH, Frixen UH, Behrens J, Unger A, Jahnke K and Birchmeier W (1991) E-Cadherin expression in squamous cell carcinomas of the head and neck: inverse correlation with tumour dedifferentiation and lymph node metastasis. *Cancer Research* **51**: 6328–6337

Shimoyama Y, Nagafuchi A, Fujita S, Gotoh M, Takeichi M, Tsukita S and Hirohashi S (1992) Cadherin dysfunction in a human cancer cell line: possible involvement of loss of α-catenin expression in reduced cell-cell adhesiveness. *Cancer Research* **52**: 5770–5774

Siitonen SM, Kononen JT, Helin HJ, Rantala IS, Holli KA and Isola JJ (1996) Reduced E-Cadherin expression is associated with invasiveness and unfavourable prognosis in breast cancer. *Am J of Cli Patho* **105**: 394–402

Van der Wurff AAM, Kate JT, Van der Linden EPM, Dinjens WNM, Arends J-W and Bosman FT (1992) L-CAM expression in normal. Premalignant and malignant colon mucosa. *J of Pathol* **168**: 287–291

*© 2001 Cancer Research Campaign*
